# Muscle-specific Bet1L knockdown induces neuromuscular denervation, motor neuron degeneration, and motor dysfunction in a rat model of familial ALS

**DOI:** 10.3389/fnins.2025.1527181

**Published:** 2025-01-17

**Authors:** Adam Eckardt, Charles Marble, Bradley Fern, Henry Moritz, Charles Kotula, Jiayi Ke, Clarisse Rebancos, Samantha Robertson, Hiroshi Nishimune, Masatoshi Suzuki

**Affiliations:** ^1^Department of Comparative Biosciences, University of Wisconsin-Madison, Madison, WI, United States; ^2^Neurobiology of Aging, Tokyo Metropolitan Institute for Geriatrics and Gerontology, Tokyo, Japan; ^3^Stem Cell and Regenerative Medicine Center, University of Wisconsin-Madison, Madison, WI, United States

**Keywords:** amyotrophic lateral sclerosis (ALS), Bet1L, small interfering RNA (siRNA), SOD1^G93A^ rats, neuromuscular junction, skeletal muscle, motor neuron, distal axonopathy

## Abstract

Amyotrophic lateral sclerosis (ALS) is a neuromuscular disease characterized by specific loss of motor neurons in the spinal cord and brain stem. Although ALS has historically been characterized as a motor neuron disease, there is evidence that motor neurons degenerate in a retrograde manner, beginning in the periphery at the neuromuscular junctions (NMJs) and skeletal muscle. We recently reported a vesicle trafficking protein Bet1L (Bet1 Golgi Vesicular Membrane Trafficking Protein Like) as a new molecule possibly linked to NMJ degeneration in ALS. In this study, we tested the hypothesis that Bet1L gene silencing in skeletal muscle could influence NMJ integrity, motor neuron function, and survival in a rat model of familial ALS (SOD1^G93A^ transgenic). Small interfering RNA (siRNA) targeting the Bet1L gene was injected on a weekly basis into the hindlimb muscle of pre-symptomatic ALS and wild-type (WT) rats. After 3 weeks, intramuscular Bet1L siRNA injection significantly increased the number of denervated NMJs in the injected muscle. Bet1L knockdown decreased motor neuron size in the lumbar spinal cord, which innervated the siRNA-injected hindlimb. Impaired motor function was identified in the hindlimbs of Bet1L siRNA-injected rats. Notably, the effects of Bet1L knockdown on NMJ and motor neuron degeneration were more significant in ALS rats when compared to WT rats. Together, Bet1L knockdown induces denervation of NMJs, but also this knockdown accelerates the disease progression in ALS. Our results provide new evidence to support the potential roles of Bet1L as a key molecule in NMJ maintenance and ALS pathogenesis.

## 1 Introduction

Amyotrophic lateral sclerosis (ALS) is a fatal neuromuscular disease in which patients gradually become paralyzed due to loss of motor neurons in the spinal cord and brainstem, leading to motor dysfunction (Brown and Al-Chalabi, 2017; Oskarsson et al., 2018; Mead et al., 2023). About 90% of ALS cases are considered sporadic, with only 10% accounted for by genetically inherited mutations in a variety of genes such as SOD1, C9ORF72, TARDBP, and FUS (Corcia et al., 2019). The pathogenesis of sporadic ALS remains largely unknown. To date, multiple mechanisms have been proposed for motor neuron degeneration, including glutamate excitotoxicity, neuroinflammation, mitochondrial dysfunction, impaired proteostasis, altered RNA metabolism, defects in nuclear cytoplasmic transport, altered neurofilament function, and dysregulated axonal/vesicular transport (Butti and Patten, 2018; Krakora et al., 2012; Mejzini et al., 2019; Petrov et al., 2017).

While all ALS forms cause motor neuron cell death, there is increasing evidence that skeletal muscle and neuromuscular junctions (NMJs) are affected from early stages of the disease. Previous studies in both ALS patients (Daube, 1985; Joyce and Carter, 2013; Carvalho and Swash, 2000) and rodent models (Krakora et al., 2012; Moloney et al., 2014) indicate that the degeneration of peripheral axon NMJs occurs early and progresses toward motor neuron cell bodies in the spinal cord. This has led to the “dying back” hypothesis, suggesting that the disease begins at the peripheral structures such as neuromuscular junctions (NMJs) and skeletal muscle (Yaron and Schuldiner, 2016; Lynch et al., 2022). Distal disease processes have become of interest as potential therapeutic targets for ALS (Krakora et al., 2012; Shefner et al., 2023; Duranti and Villa, 2023), but the specific processes involved in skeletal muscle and the NMJs are still largely unknown.

To explore possible contributions of skeletal muscle to ALS pathogenesis, we recently performed transcriptome analysis of skeletal myocytes prepared from patient-derived induced pluripotent stem cell lines with familial or sporadic ALS backgrounds (Lynch et al., 2021). RNA sequencing revealed that four genes, *BET1L, GPC3, DCX*, and *HNRNPK*, were commonly downregulated when compared to control myocytes (Lynch et al., 2021). Among the four identified molecules, our current focus is the vesicle trafficking protein Bet1L (Bet1 Golgi Vesicular Membrane Trafficking Protein Like). Bet1L gene expression was commonly downregulated in the limb muscle of a rat model of familial ALS (SOD1^G93A^ transgenic) (Howland et al., 2002; Suzuki et al., 2007b; Hayes-Punzo et al., 2012), with decreased expression over time following disease progression (Lynch et al., 2021). Furthermore, immunohistochemistry revealed that Bet1L protein was focally localized in the basal lamina of the NMJ (Lynch et al., 2021). Bet1L (also known as GS15) has been known to be involved in retrograde vesicle-mediated transport, Golgi-related vesicle transport, and enabling SNAP receptor activity as a component of the SNARE protein complex (Xu et al., 2002; Tai et al., 2004). However, the specific functions of Bet1L have not been fully elucidated.

Loss of Bet1L at the NMJ could be of interest for better understanding ALS disease progression as well as the biological roles of this molecule. In this study, our working hypothesis is that the reduction of Bet1L levels in skeletal muscle, specifically at NMJs, influences the NMJ integrity, motor neuron degeneration, and motor dysfunction in a rat model of familial ALS (SOD1^G93A^ transgenic rats). The overexpression of mutant SOD1 in rodents causes specific characteristics in pathology similar to those seen in human ALS patients (Zhu et al., 2023; Gurney, 1994). To test our hypothesis, we proposed to knock down Bet1L gene expression in the skeletal muscle by small interfering RNA (siRNA). siRNA can suppress the expression of a specific gene of interest by degrading mRNA after transcription and preventing translation (Elbashir et al., 2001; Lagana et al., 2014; Deogharia and Gurha, 2022; Khan et al., 2023). It is a class of double-stranded RNA, typically 20–24 (normally 21) base pairs in length, that acts in the RNA interference pathway by interfering with the expression of genes with complementary nucleotide sequences to that of the siRNA. In this study, siRNA targeting Bet1L was prepared and delivered into the hindlimb muscles of wild-type or SOD1^G93A^ transgenic rats. Immunohistochemistry, locomotor function tests, and motor neuron counting were then performed to quantify changes in NMJ denervation, motor neuron degeneration, and motor dysfunction in Bet1L siRNA-injected rats.

## 2 Materials and methods

### 2.1 Bet1L siRNA preparation

Pre-designed rat Bet1L siRNA was obtained from GE Healthcare Dharmacon (L-091291-02-0005, Chicago, IL). Scrambled sequence siRNA (Dharmacon, D-001810-10-05) was also purchased as a control. siRNA was mixed with Oligofectamine^TM^ transfection reagent (12252-011, Thermo Fisher Scientific, Waltham, MA) and RNAase (Qiagen, Hilden, Germany) in a buffer (diluted from 5 × siRNA buffer, NC1338268, Thermo Fisher Scientific) for a final concentration of 5 μM siRNA. Oligofectamine is a fast-acting, non-toxic transfection reagent that has been used to deliver siRNA into nuclear or cytoplasmic targets both *in vitro* and *in vivo* (Nakajima et al., 2012; Seven et al., 2018).

### 2.2 SOD1^*G*93*A*^ transgenic rats

All the animal procedures in the present study were approved by the University of Wisconsin School of Veterinary Medicine Animal Care and Use Committee (IACUC, Protocol ID V005430) and carried out in accordance with the guidelines for our IACUC and NIH standards of animal care. SOD1^G93A^ transgenic and age-matched wild-type rats were used in this study. SOD1^G93A^ transgenic male founders, originally obtained from Taconic (Hudson, NY) (Howland et al., 2002), were crossed with wild-type female Sprague-Dawley rats to maintain colonies. This rat colony was maintained as previously described (Suzuki et al., 2007b; Hayes-Punzo et al., 2012; Van Dyke et al., 2016; Lynch et al., 2021). All rats were housed, bred, and sacrificed in accordance with UW-Madison and NIH standards of animal care. Genotyping of SOD1^G93A^-positive rats was determined from ear punches using real-time PCR, which was performed by Transnetyx (Cordova, TN). Based on our latest analysis, the recent colonies of our ALS rats showed disease onset on average at day 179.5 ± 8 days (Lynch et al., 2021). The ALS rats used in this study were expected to be at the pre-symptomatic stage because all experiments were concluded before 96 days of age (Suzuki et al., 2007b; Hayes-Punzo et al., 2012; Lynch et al., 2021).

### 2.3 Intramuscular siRNA injection

In the first study, we used healthy wild-type rats and evaluated the efficacy of Bet1L siRNA for gene silencing. The treatment groups were: (1) *Bet1L* siRNA, (2) scrambled siRNA, (3) vehicle with Oligofectamine, and (4) no injection (sham control). Animals were anesthetized with isoflurane, and then treatment solutions (30 μL per muscle) were injected unilaterally into tibialis anterior (TA) muscles of WT rats. The injections were repeated for 3 days. The subsequent injections were performed nearly at the same site as the original location of the muscle, slowly releasing siRNA or vehicle. On the 4th day, the animals were sacrificed, and their hindlimb muscles (tibialis anterior muscle, TA) were collected. The non-injected TA muscles from the unilaterally Bet1L siRNA animals were used in analysis as the no-injection treatment group.

**Figure 1:**
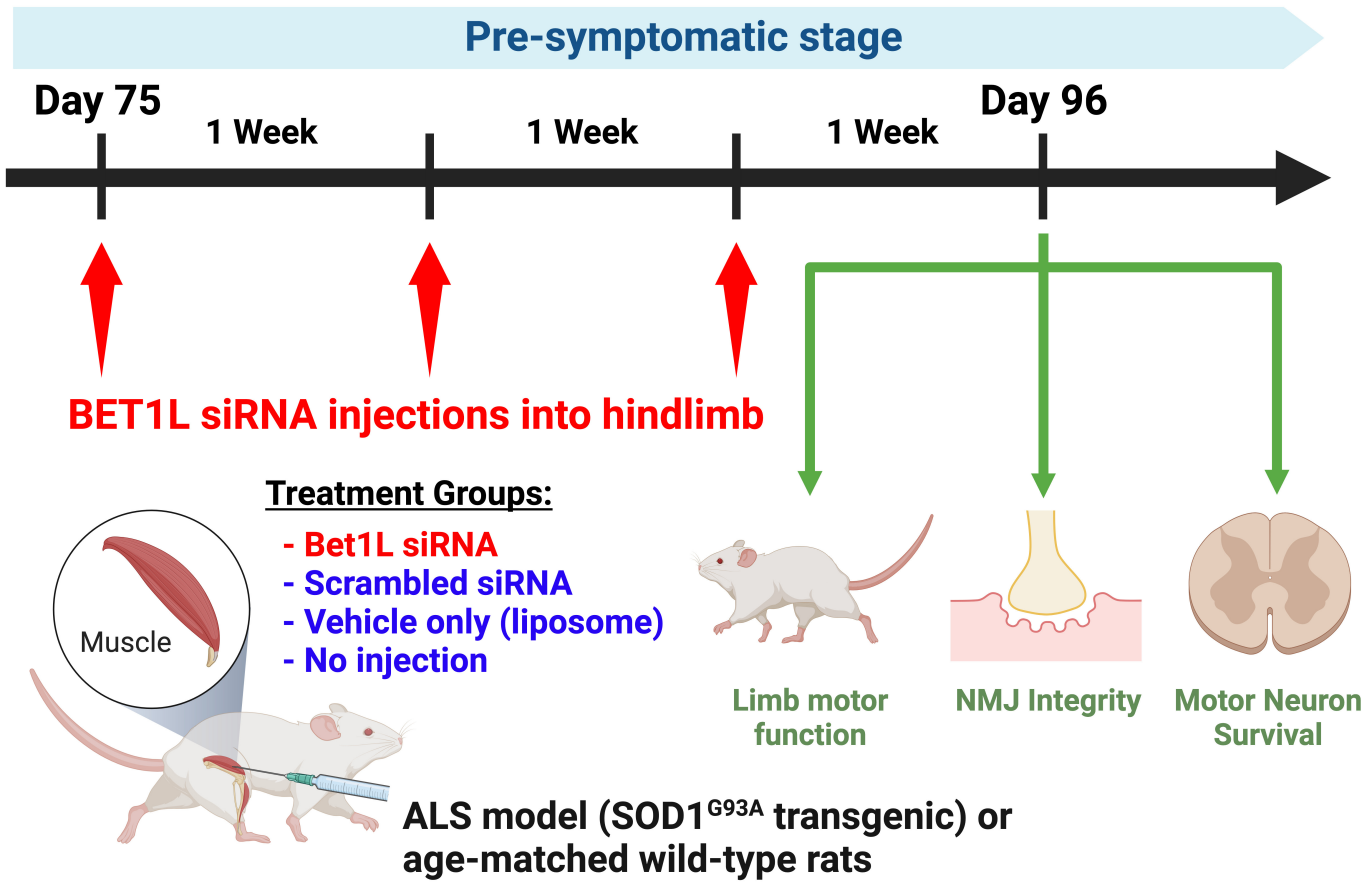
*In vivo* Bet1L gene silencing by intramuscular small interfering RNA (siRNA) injection in ALS model or wild-type (WT) rats. Small interfering RNA (siRNA) targeting the Bet1L gene was injected weekly into the hindlimb muscle of pre-symptomatic ALS rats (SOD1^G93A^ transgenic) starting at 75 days of age. Four treatment groups were prepared: Bet1L siRNA, scrambled siRNA, vehicle, and no injection (sham). Treatment solutions (30 μL per muscle) were unilaterally injected into the hind limb muscles (TA, gastrocnemius, and quadriceps femoris muscles). The same treatment groups were also prepared with age-matched WT rats to identify how the transgenic background influenced the effects of Bet1L siRNA injection. The motor function in the siRNA-injected limb was monitored once per week by the Basso Beattie and Bresnahan (BBB) locomotor rating scale. After 3 weeks (i.e., 96 days old), rats were sacrificed and processed for neuromuscular junction (NMJ) innervation in the siRNA-injected muscle and motor neuron survival in the lumbar spinal cord. This scheme represents the studies in Figures 4–6, while the samples used in Figures 2, 3 were collected in the 1st week of the injection (on the 4th day after 3 consecutive days of injection).

In the subsequent experiments, pre-symptomatic SOD1^G93A^ rats received intramuscular injections at 75 days of age (Figure 1). Four treatment groups were prepared (Bet1L siRNA, scrambled siRNA, vehicle, and no injection). Similar treatment groups of age-matched WT rats were also prepared. Treatment solutions (30 μL per muscle) were unilaterally injected into the hind limb muscles (TA, gastrocnemius, and quadriceps femoris muscles). The injections were repeated 3 days in a row. After 1 week (i.e., 82 days of age) or 3 weeks (96 days of age) after the first injection, TA muscles and spinal cords were collected from each rat). The non-injected TA muscles of the unilaterally Bet1L siRNA animals were used in analysis as the no-injection treatment group.

### 2.4 Muscle tissue homogenate preparation

Muscle tissues (~200–250 mg) were homogenized using a Silent Crusher M tissue homogenizer (Heidolph, Wood Dale, IL) in 1 mL of ice-cold homogenization buffer (20 mM Tris-Cl pH 7.8, 137 mM NaCl, 2.7 mM KCl, 1 mM MgCl_2_, 1 mM EDTA, 1 mM DTT, 1% Triton X-100, 10% glycerol; all from Sigma Aldrich) supplemented with Halt Protease Inhibitor Single-use Cocktail (100 × , 78430, Thermo Scientific, Rockford, IL). Protein concentrations were determined using the Pierce BCA Protein Assay Kit (23227, Thermo Scientific).

### 2.5 Western blotting

Muscle tissue homogenates (20 μg protein per lane) were run on a 12.5% polyacrylamide gel and transferred onto a PVDF membrane (Millipore Sigma, Burlington, MA). The membrane was immunoblotted with anti-Bet1L (1:1000, mouse monoclonal, BDB610960, BD Biosciences, Franklin Lakes, NJ) or anti-glyceraldehyde 3-phosphate dehydrogenase (GAPDH) antibodies (1:1000, rabbit monoclonal, D16H11, Cell Signaling Technology Inc., Danvers, MA). Following secondary incubation with anti-mouse or rabbit peroxidase-tagged antibody (1:1000, Promega, Madison, WI) and incubation with enhanced chemiluminescence (Pierce ECL Western Blotting Substrate, Thermo Fisher Scientific), the membrane was scanned by the LI-COR Odyssey XF imaging system (Lincoln, NE). The protein band signals were densitometrically analyzed using ImageJ software.

### 2.6 Neuromuscular junction (NMJ) immunohistochemistry

TA muscles were transversely sectioned at 20-micron thickness using a cryostat. Muscle sections spanned ~920 mm from the injection site (where the injected reagents had been distributed). The muscle sections were placed on a glass slide and fixed with 4% paraformaldehyde in phosphate-buffered saline (PBS) for 20 min at room temperature. Sections were then blocked in 5% normal donkey serum (NDS, Jackson ImmunoResearch Labs, West Grove, PA) in PBS for 2 h at room temperature, followed by primary antibody incubation in 5% NDS and 0.3% Triton-X 100 (Sigma Aldrich) overnight at 4°C. Then the sections were washed five times for 5 min each with PBS before adding secondary antibodies diluted in PBS supplemented with 5% NDS. After the wash step was repeated, some sections were incubated in Hoechst 33258 (1:10,000 in PBS, Sigma Aldrich) for 15 min at room temperature, followed by additional washes. Fluoromount-G mounting medium (Thermo Fisher Scientific) was applied to the slide after the last wash, and a glass coverslip was placed on top. Bet1L expression at the NMJs was identified by co-labeling with α-bungarotoxin (BTX) conjugated to Alexa Fluor 647 or 594 (1:1,000, Thermo Fisher Scientific) and an anti-Bet1L antibody (1:1,000, rabbit polyclonal, PA5-58943, Thermo Fisher Scientific). To determine any correlations between Bet1L expression and NMJ innervation, the degree of NMJ integrity was determined by co-staining for Bet1L, motor endplate (BTX), and motor axon terminals labeled by two antibodies against synaptic proteins and neurofilaments (1:40 with SV2 and 2H3 clones, respectively, mouse monoclonal, Developmental Studies Hybridoma Bank, Iowa City, IA).

For some images to secure their entire NMJ orientation, a modified immunostaining protocol was applied to longitudinal muscle sections (Nishimune et al., 2008; Rogers et al., 2017). Briefly, TA muscles were fixed with 2% PFA-PBS for 30 min at room temperature, rinsed in PBS a few times, and submerged in 20% sucrose in PBS for over 48 h. Fixed muscles were frozen in Optimal cutting temperature compound (Fisher Scientific) and longitudinally cut at 20-micron thickness using a cryostat. After processing with similar staining steps with antibody incubations and washing as described above, a cover slide was mounted on the slide glass with Prolong Glass Antifade Mountant (Thermo Fisher Scientific).

### 2.7 NMJ image acquisition and analysis

Fluorescent images were taken using a Nikon Eclipse 80i fluorescent microscope (Nikon Instruments Inc., Tokyo, Japan) with an ORCA-Fusion BT digital camera (C15440, Hamamatsu Photonics, Shizuoka, Japan). For some representative images, the three-dimensional renderings of rat NMJ immunohistochemistry were created using the z-stack feature of a Leica TCS SP8 confocal microscope (Leica, St. Galen, Switzerland). The number of Bet1L-positive or negative NMJs was quantified for each animal as an average from four different muscle sections. The innervation rate analysis of NMJs has been described previously (Rogers et al., 2017; Lynch et al., 2021). Briefly, fully innervated NMJs were identified by perfect overlap with adult motor nerve terminals and acetylcholine receptor clusters, which were identified by the antibodies against motor axon terminals (synaptic protein SV2A and neurofilament NH-M, SV2 + NF) and acetylcholine receptor (AChR)-positive endplates in the muscle (Rogers et al., 2017). NMJs were assessed for areas of the acetylcholine receptor clusters that were not occupied by nerves, whether in part or in full, which were considered to be denervated NMJs (Rogers et al., 2017). The average number of total NMJs counted per animal was 60–80. Quantifications were performed from 4 to 7 rats of each experimental group, leading to ~350 NMJs counted per group. When analyzing the number of immunolabeled NMJs, the researchers performing the quantification were blinded to the treatment group, and results were averaged between them to avoid potential bias. The results were then confirmed by multiple lab staff.

### 2.8 Motor neuron analysis

We performed motor neuron analysis similarly as described in previous papers (Suzuki et al., 2007a, 2008, 2010; Krakora et al., 2013; Austin et al., 2022). Briefly, small tissue segments of rat spinal cord (T13-L6) were embedded in paraffin, sectioned by a Leica RM2255 microtome, and processed for cresyl violet staining. Previous studies demonstrated that the T13-L6 segments of the spinal cord innervate the siRNA-injected hindlimb muscles (Suzuki et al., 2007a; Mohan et al., 2014, 2015). Bright-field microscopy images were captured from the ventral horn of the spinal cord using a Keyence BZ-X710 microscope (Osaka, Japan). A standardized circular area of interest was placed over the ventral horn of the spinal cord image, and a scale bar was added in Google Draw (San Francisco, CA). Nissl-stained motor neurons residing in the area of interest were characterized by a distinct nucleolus and a darkly stained cytoplasm. The diameter (an average of the minimal and maximal diameter) and average number of motor neurons were analyzed by NIH ImageJ software. As in the NMJ quantification above, the analysis was blindly performed by multiple lab staff.

### 2.9 Motor function assessment

For the ALS rats that received intramuscular siRNA injection for 3 weeks, changes in hindlimb motor function were assessed weekly in a small enclosure using the Basso, Beattie, and Bresnahan (BBB) locomotor rating scale (Basso et al., 1995). The BBB locomotor rating scale was obtained as previously described (Suzuki et al., 2007b; Hayes-Punzo et al., 2012). Each rat was allowed to walk around in an open cage while we observed hindlimb movements for ~3–5 min. Each hind limb score was based on the 21-point scoring scale from no movement (0) to normal locomotion (21). Pre-symptomatic was defined as ranging from 21 to 18, symptom onset from 17 to 14, and symptomatic from 13 to 0 (Suzuki et al., 2007b; Hayes-Punzo et al., 2012). Scoring criteria include paw rotation, toe clearance, weight support, the frequency of each abnormality to locomotion, and the amount of movement occurring from each joint (Basso et al., 1995). To avoid potential bias, the BBB scaling was performed in a blinded manner with regard to the treatment group. Additionally, video clips were recorded from each rat and used to confirm the results by three different lab staff.

### 2.10 Statistical analysis

GraphPad Prism 9.4.0 (GraphPad Software, Inc., La Jolla, CA) was used to perform statistical analyses. Quantitative data were graphed as means ± standard error of the mean (SEM) from at least two independent experiments. One-way analysis of variance (ANOVA) was used to compare data in the same animal group. A statistically significant one-way ANOVA was followed up with Tukey's *post-hoc* test for multiple comparisons. A two-way ANOVA was calculated to compare multiple groups, followed up with Tukey's multiple comparisons test. Differences were considered significant when *P* < 0.05.

## 3 Results

### 3.1 Bet1L siRNA sufficiently reduced Bet1L expression at the NMJs in wild-type rats

**Figure 2:**
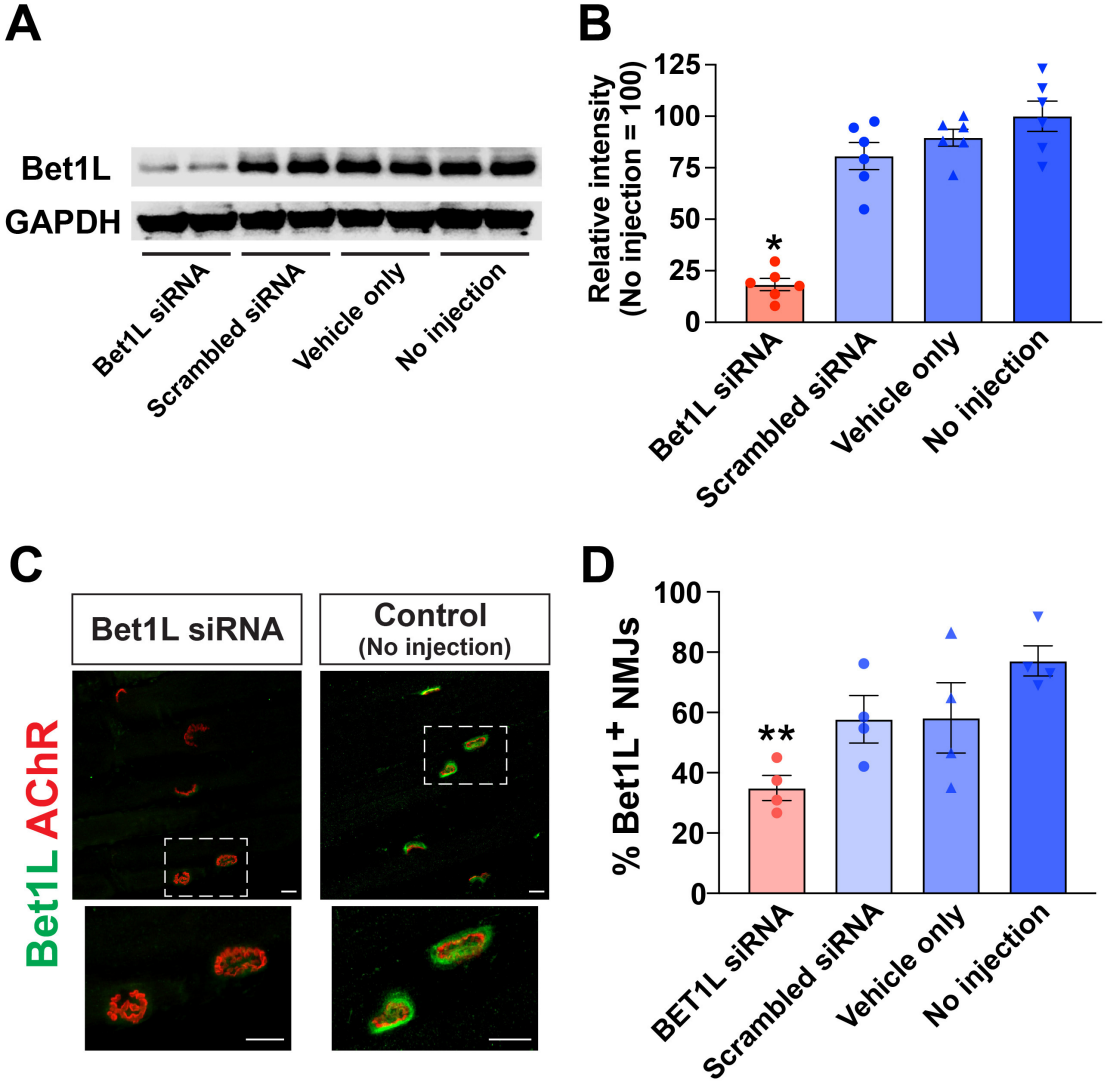
Small interfering RNA (siRNA)-mediated knockdown of BET1L was efficacious in WT rats. **(A)** A representative Western blot image showing the relative levels of Bet1L protein in using muscle homogenates. Bet1L siRNA was prepared at the final concentration of 5 mM and injected into the hind limb (tibialis anterior) muscles. Three control groups were prepared: scrambled siRNA, vehicle only (Oligofectamine), and no-injection sham. Glyceraldehyde 3-phosphate dehydrogenase (GAPDH) was used as an internal control. In this electrophoresis, two muscle samples from different rats were loaded for each treatment group. **(B)** The densitometric analysis of proteins revealed that Bet1L siRNA injection significantly reduced its protein level up to ~18% when compared to the level in the non-injection control rats. *P < 0.05 vs. three control groups, *n* = 6 each. **(C)** Representative images of immunohistochemistry showing the decreased Bet1L protein at the neuromuscular junctions (NMJs) in the Bet1L siRNA-injected muscle. AChR, acetylcholine receptor. Scale bar = 10 mm. **(D)** The number of Bet1L-positive NMJs was significantly decreased after 1 week of Bet1L siRNA injection in WT rats. **P < 0.01 vs. three control groups, *n* = 4 each.

In order to explore the effects of Bet1L siRNA-based knockdown, we first confirmed siRNA efficacy for gene silencing using healthy wild-type rats. Bet1L siRNA was prepared at a final concentration of 5 mM and injected into the tibialis anterior (TA) muscles of healthy adult WT rats. Three control groups were prepared: scrambled siRNA, vehicle only (Oligofectamine), and no-injection sham. After the injections were repeated for 3 days, the animals were sacrificed on the 4th day, and the injected TA muscles were collected. We performed Western blotting using muscle homogenates (Figure 2A). The densitometric analysis of proteins revealed that Bet1L siRNA injection significantly reduced its protein level up to ~18% (*P* < 0.05, 18.3 ± 2.8%) when compared to the level in the other three controls (80.7 ± 6.5% in scrambled siRNA, 89.7 ± 4.1% in vehicle only, 100.0 ± 7.3% in no injection; *n* = 6 in each group) (Figure 2B).

Next, we performed immunohistochemistry to identify siRNA-based inhibition of Bet1L levels at the NMJs in the siRNA-injected muscle (Figures 2C, D). The overlap of acetylcholine receptor (AChR)-positive motor endplates (labeled by fluorochrome-conjugated BTX) and Bet1L proteins indicated the presence of Bet1L at the NMJs in the control rats. In contrast, the lack thereof depicted the reduction or absence of Bet1L in the Bet1L siRNA-injected muscle (Figure 2C). Corresponding to our recent observations (Lynch et al., 2021), confocal microscopy using longitudinal muscle sections showed the specific localization of Bet1L protein at the NMJ of a no-injection control rat (Supplementary Movie 1). However, Bet1L expression was reduced at the NMJ by intramuscular Bet1L siRNA injection (Supplementary Movie 2). After quantitative comparison between four experimental groups (*n* = 4 each), we found that Bet1L siRNA-injected muscles had a lower percentage of NMJs positive with Bet1L when compared to three control groups (Figure 2D). Specifically, the difference was statistically significant between Bet1L siRNA-injected and the non-injection control (*P* < 0.01) (Figure 2D).

### 3.2 Bet1L siRNA-based gene silencing induced NMJ denervation in WT and ALS rats

**Figure 3:**
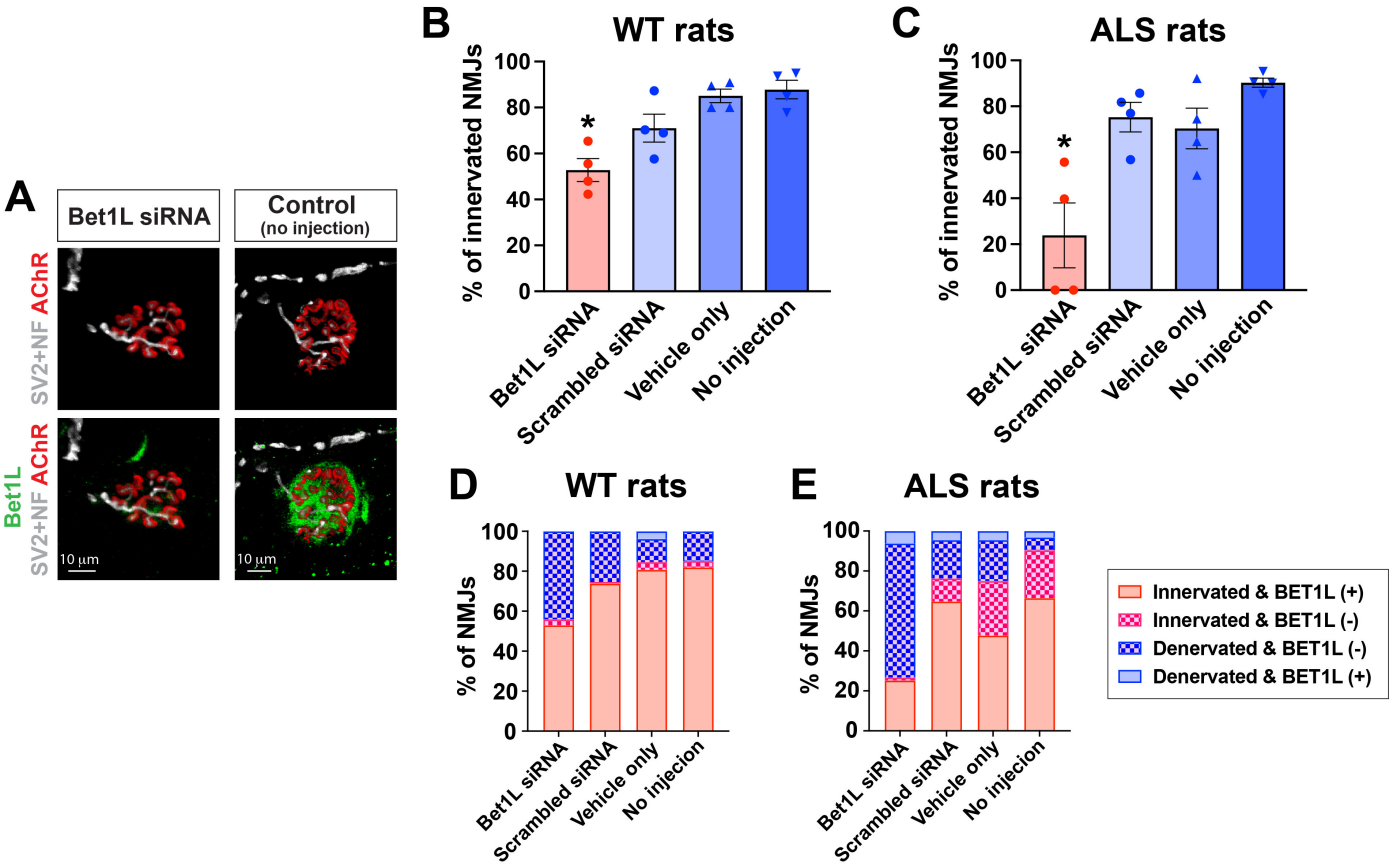
Intramuscular Bet1L siRNA injection significantly decreased innervation in the skeletal muscle of WT and pre-symptomatic ALS rats. **(A)** Representative images of immunohistochemistry for individual NMJs in Bet1L siRNA-treated and control (no injection) rats. After 1 week of siRNA injection, NMJ integrity was identified by co-staining with Bet1L, motor axon terminals (synaptic protein SV2A and neurofilament NF-M, SV2 + NF), and acetylcholine receptor (AChR)-positive endplates. **(B, C)** Quantification of NMJ innervation in WT **(B)** and ALS **(C)** rats. *p < 0.05 vs. all three control groups. *n* = 4 for each group. **(D, E)** Correlation of NMJ innervation and Bet1L expression. Similarly, as observed in both WT **(D)** and ALS **(E)** rats, the relationship between NMJ integrity and Bet1L expression at NMJs indicated that innervated NMJs expressed Bet1L.

After confirming the ability of Bet1L siRNA to reduce Bet1L expression in rat skeletal muscle, we next determined whether distal morphological changes could occur by silencing Bet1L expression. WT rats at 75 days of age were unilaterally injected with their respective treatment (Bet1L siRNA, scrambled siRNA, vehicle only, or sham) into their TA muscles (*n* = 4). The injection was repeated daily for 3 days. At 4 days after the initial injection, the TA muscles were collected and sectioned to determine both NMJ innervation and Bet1L expression at the NMJs by immunohistochemistry (Figures 3A, B). A quantitative analysis of NMJ innervation showed that intramuscular Bet1L siRNA injection significantly decreased the percentage of innervated NMJs when compared to all three controls (Figure 3B). Corresponding to these innervation results, the percentage of denervated NMJs was increased in Bet1L siRNA-injected rats (Figure 3B).

Next, we accounted for the potential impact of the ALS genetic background on the morphological outcome of silencing Bet1L expression. Similarly to the last study using WT rats, presymptomatic ALS rats (75 days old) were unilaterally injected with their respective treatment (Bet1L siRNA, scrambled siRNA, vehicle only, or sham; *n* = 4 each group) into their TA muscles, and we analyzed NMJ innervation and Bet1L expression by immunohistochemistry (Figure 3C). The knockdown of Bet1L in ALS rat muscles significantly reduced NMJ integrity (23.9 ± 14.2%), decreasing the percentage of innervated NMJs (Figure 3C).

We further analyzed the correlation between NMJ integrity and Bet1L expression following Bet1L siRNA-based gene silencing (Figures 3D, E). In both WT and ALS rats with Bet1L siRNA treatment, the majority of innervated NMJs had Bet1L present, whereas the majority of denervated NMJs did not express Bet1L. Similar correlations between Bet1L expression and NMJ integrity were observed in all three control groups.

### 3.3 Prolonged knockdown of Bet1L expression also induced NMJ denervation in pre-symptomatic ALS rats

Next, we prepared another cohort of pre-symptomatic ALS and age-matched WT rats and unilaterally injected Bet1L siRNA in their hindlimb muscles (TA, gastrocnemius, and quadriceps femoris muscles). The animals received the injection once per day for the first 3 days in a week, and these weekly injections were repeated for up to 3 weeks. We then observed the animals closely for any changes to motor neuron function and viability from prolonged Bet1L knockdown.

**Figure 4:**
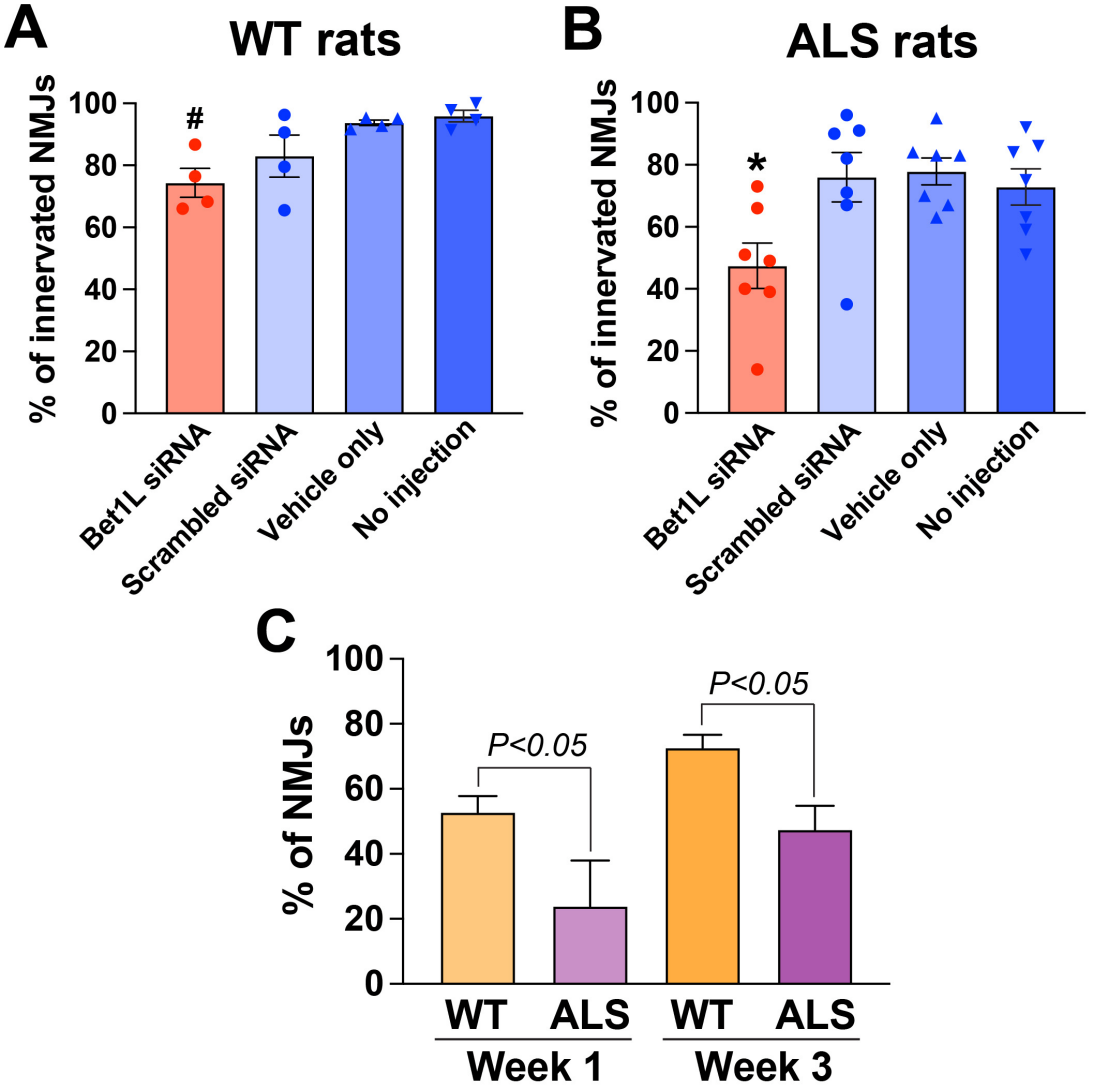
A significant reduction of NMJ innervation was confirmed in rat muscles following intramuscular Bet1L siRNA injection as repeated for 3 weeks. A new cohort of WT **(A)** and pre-symptomatic ALS rats **(B)** (75 days of age) were unilaterally injected with Bet1L siRNA in their hindlimb muscles and received the injection every week (as outlined in Figure 1). In the third week after the first siRNA injection, NMJ innervation/denervation in TA muscles was analyzed by immunohistochemistry. ^#^*p* < 0.05 vs. vehicle only and no injection. *p < 0.05 vs. all three controls. **(C)** By Bet1L siRNA injection, more severe effects in NMJ degeneration were identified in ALS model rats when compared to WT rats. The statistical difference was calculated by a two-way ANOVA followed by Tukey's multiple comparisons test.

At the end of the experiment, in the third week after the first siRNA injection, TA muscles were harvested, sectioned, and co-stained to analyze any changes in NMJ innervation. In WT rats (Figure 4A), intramuscular siRNA injection for 3 weeks significantly decreased the percentage of innervated NMJs (*P* < 0.05, 74.4 ± 4.7%) when compared to two control groups, vehicle (93.8 ± 0.9%) and no injection (96.0 ± 1.9%, *n* = 4 each group). There was a similar trend in the difference between Bet1L and scrambled siRNA-treated rats, but the difference did not reach significance. Bet1L siRNA-injected ALS rats had significantly lower levels of innervated NMJs (*P* < 0.05, 47.4 ± 7.3%) when compared to all three controls (76.0 ± 7.9% scrambled, 77.9 ± 4.3% vehicle, and 72.9 ± 5.8% no injection, *n* = 7 each group; Figure 4B).

Together with the results in Figure 3, we calculated differences in the effect of Bet1L gene silencing in NMJ innervation using two-way ANOVA in terms of the genotype (WT vs. ALS), duration (Week 1 vs. Week 3), and their interaction in Bet1L siRNA-treated animals (Figure 4C). While there was no statistical difference in the duration, a significant difference was observed in the genotype (*P* < 0.001). The interaction between genotype and duration was not significantly different. Additional *post-hoc* comparisons revealed that ALS rats showed a lower level of innervated NMJs at both Week 1 and Week 3 (*P* < 0.05, Figure 4C), indicating that more severe effects in NMJ integrity were induced by siRNA-based Bet1L in ALS model rats. In contrast, a similar statistical analysis did not identify any statistical differences in the three control groups.

### 3.4 Bet1L siRNA-based knockdown in skeletal muscle influenced motor neuron survival

**Figure 5:**
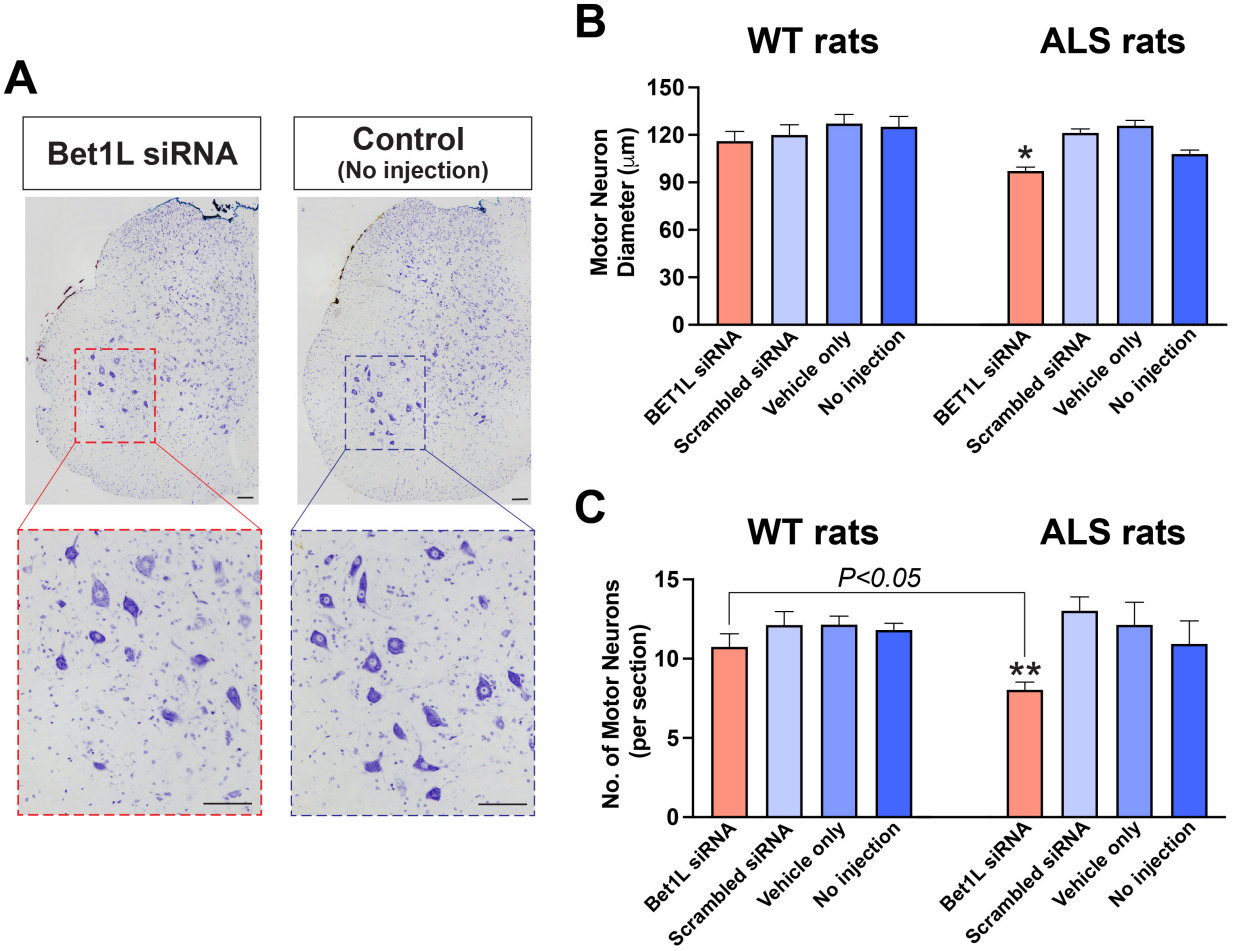
Intramuscular Bet1L siRNA injection affected motor neuron survival within the lumbar spinal cord region that projected to the injected muscles. **(A)** Representative images of cresyl violet staining showing the large motor neurons in the lumbar spinal cord sections in Bet1L siRNA-treated and control ALS rats. The diameter **(B)** and number **(C)** of motor neuron cell bodies were analyzed in the ventral horn of the lumbar spinal cord. In **(C)**, only motor neurons having large cell bodies (approximately over 80 μm in diameter) were counted. Scale bar = 100 mm in **(A)**. *p < 0.05 vs. all three controls; **p < 0.05 vs. scrambled siRNA.

We next hypothesized that the distal denervation at the NMJs, which were induced by intramuscular Bet1L siRNA injection, would lead to motor neuron degeneration. To answer this, we prepared spinal cord sections from the animals used for NMJ analyses above and analyzed cresyl violet (Nissl)-stained motor neurons within the region (T13-L6) that innervated the siRNA-injected hindlimb muscles (Suzuki et al., 2007a; Mohan et al., 2014, 2015) (Figure 5A).

We first determined the diameter of motor neuron cell bodies (the average of the minimal and maximal diameter) in the ventral horn of the spinal cord (Figure 5B). In WT rats, the diameter of motor neuron cell bodies was commonly lower in Bet1L siRNA-injected rats (116.0 ± 6.1 mm, *n* = 63) when compared to scrambled siRNA (119.8 ± 6.5 mm, *n* = 67), vehicle only (127.1 ± 5.7 mm, *n* = 61), and no injection (125.2 ± 6.5 mm, *n* = 58). However, this difference did not reach significance (Figure 5B left). In contrast, the cell diameter of motor neurons in Bet1L siRNA-injected ALS rats (73.3 ± 1.9 mm, *n* = 140, *P* < 0.05) was significantly lower than all controls: 91.6 ± 1.8 mm in scrambled siRNA (*n* = 91), 94.9 ± 2.6 mm in vehicle only (*n* = 85), and 81.4 ± 1.9 mm in no injection (*n* = 126) (Figure 5B right).

Next, the number of motor neurons was determined using the spinal cord sections (Figure 5C). We counted the neurons with large-sized cell bodies in the ventral horn, which represent alpha motor neurons. In WT rats (Figure 5C left), there was no significant difference in the number of motor neurons between the four treatment groups: 10.7 ± 0.8 in Bet1L-siRNA, 12.1 ± 0.8 in scrambled siRNA, 12.1 ± 0.8 in vehicle-only, and 12.1 ± 0.8 in non-injection (*n* = 4 each). In ALS rats, the number of alpha motor neurons was lower in the Bet1L siRNA-injected rats (8.0 ± 0.5; *n* = 7) when compared to scrambled siRNA-treated rats (13.1 ± 0.9, *n* = 7; *P* < 0.05) (Figure 5C right). The two other controls also showed a similar trend (vehicle only 12.1 ± 1.4; no injection 10.9 ± 1.5; *n* = 7 each), although the difference was not statistically significant.

Furthermore, we analyzed differences in the effect of Bet1L gene silencing by comparing the values between genotypes (WT vs. ALS). Although no statistical differences were identified in the motor neuron diameter, ALS rats showed a lower number of motor neurons than WT rats when Bet1L-siRNA was treated (*P* < 0.05, Figure 5C).

### 3.5 Bet1L knockdown impaired locomotion capability in both WT and ALS rats

**Figure 6:**
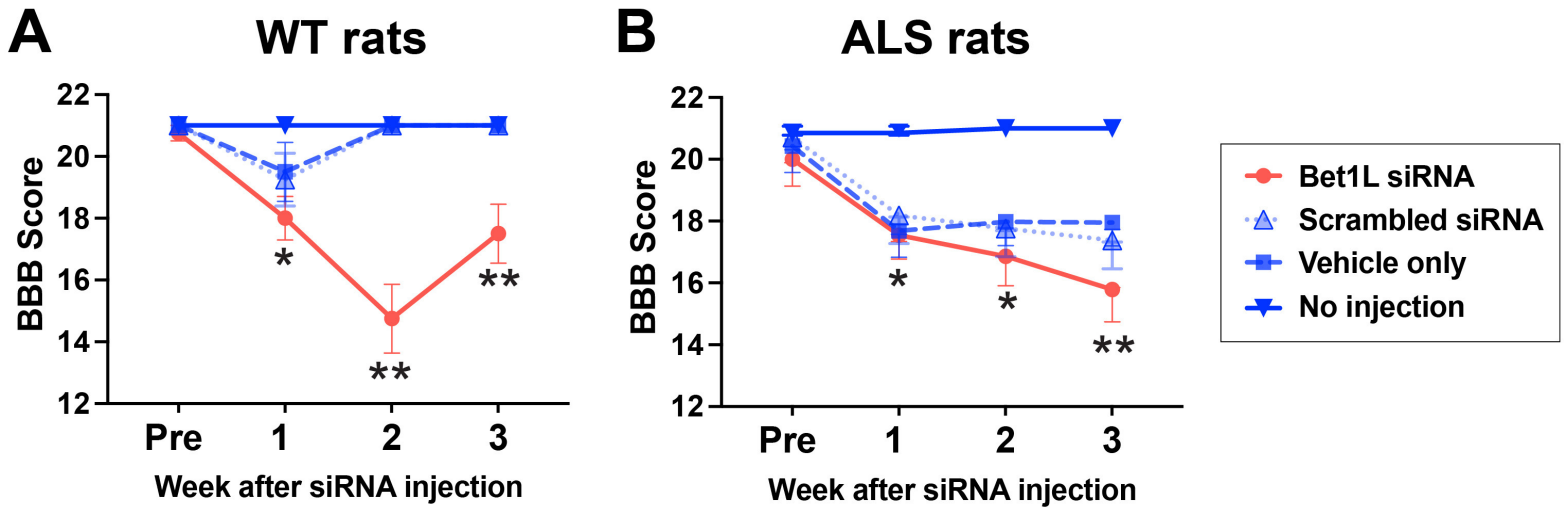
Changes of motor function in the Bet1L-injected limb of WT and ALS rats. The Basso, Beattie, and Bresnahan (BBB) locomotor scale was used to analyze the motor function of the injected hindlimbs of WT **(A)** and ALS rats **(B)**. *P < 0.01 vs. no injection, **P < 0.01 vs. all three controls. *n*=4 in WT rats and *n* = 7 in ALS rats.

Over the experimental period, individual limb function had been analyzed using the Basso-Beattie-Bresnahan (BBB) rating test (Basso et al., 1995) (Figure 6). A BBB score of 21 indicated consistent plantar stepping and coordinated gait, consistent toe clearance, predominant paw position parallel throughout stance, consistent trunk stability, and a tail consistently up, whereas a BBB score of 0 indicated no observable hindlimb movement (complete paralysis) (Basso et al., 1995).

In WT rats (Figure 6A), there was no obvious difference in the locomotor function between treatment groups after 1 week of unilateral injection. The BBB score was decreased by intramuscular Bet1L siRNA injection in the second (14.8 ± 1.1) and third (17.5 ± 1.0) weeks of the injection, while all rats in the other three groups were identified as having a BBB score of 21. A two-way ANOVA revealed that there was a statistical difference in the treatment (*F*_3, 48_ = 33.16, *P* < 0.0001), week (*F*_3, 48_ = 7.478, *P* = 0.0003), and their interaction (*F*_9, 48_ = 6.534, *P* < 0.0001). Based on multiple comparisons using the *post-hoc* analysis, Bet1L siRNA-treated rats showed significantly lower BBB scores after 2 weeks when compared to the other control groups (*P* < 0.01, Figure 6A).

In ALS rats, Bet1L siRNA-based knockdown showed a similar trend. Specifically in the third week, Bet1L siRNA-treated rats significantly decreased BBB scores (15.8 ± 0.6, *P* < 0.01) when compared to three control groups (scrambled siRNA, 17.4 ± 0.4; vehicle, 18.0 ± 0.3; and no injection control, 21.0 ± 0.0) (Figure 6B). Similarly, as seen in WT rats, a two-way ANOVA identified a statistical difference in the treatment (*F*_3, 96_ = 72.49, *P* < 0.0001), week (*F*_3, 96_ = 42.66, *P* < 0.0001), and their interaction (*F*_9, 96_ = 6.45, *P* < 0.0001). Additional *post hoc* analysis found that Bet1L siRNA-treated rats significantly reduced their BBB scores after 1 week of the treatment (*P* < 0.01, Figure 6B). Although all three controls in WT rats fully recovered their motor function by the 3rd week (Figure 6A), ALS rats treated with either scrambled siRNA or the vehicle showed lower BBB scores when compared to the no-injection control (Figure 6B).

In summary, our results indicated that Bet1L siRNA-based knockdown of Bet1L influenced NMJ innervation and motor function in both WT and ALS rats. Bet1L gene silencing commonly showed more severe effects in ALS rats, indicating that the disease progression was accelerated by Bet1L siRNA injection, and the disease stage shifted from pre-symptomatic to symptomatic.

## 4 Discussion

It has been suggested that ALS may be initiated by distal degeneration at the NMJs (Fischer et al., 2004; Krakora et al., 2012; Moloney et al., 2014; Yaron and Schuldiner, 2016; Piol et al., 2023; Shefner et al., 2023; Gao et al., 2024; Kubat and Picone, 2024). For instance, morphological changes at the NMJs were identified prior to disease onset in SOD1^G93A^ mice (Fischer et al., 2004; Clark et al., 2016; Mejia Maza et al., 2021). Our current study operated under the hypothesis that the decreased expression of Bet1L at the NMJs would influence NMJ integrity, leading to motor neuron degeneration and motor dysfunction. The foundation of our hypothesis was based on our recent study using ALS patient-iPSC-derived skeletal myocytes and ALS model rats (Lynch et al., 2021).

To knock down Bet1L expression in rat skeletal muscle, we used siRNA, a type of microRNA that can induce the biological mechanism of RNA interference to selectively silence a particular gene (Elbashir et al., 2001; Pratt and Macrae, 2009; Whitehead et al., 2009; Dana et al., 2017). Once siRNA is incorporated in a cell, siRNA binds with the RNA-induced silencing complex (RISC) in the cytoplasm (Pratt and Macrae, 2009). RISC can unwind siRNA, remove the sense strand of the siRNA, and leave only the antisense siRNA sequence as the guide strand. RISC then uses the guide strand of the siRNA as a template for recognizing complementary mRNA. Through the action of the Argonaute protein (a catalytic subunit on the RISC protein complex), the bound mRNA is cleaved and degraded (Iwakawa and Tomari, 2022). The activated RISC protein complex continues to bind complementary mRNA strands, further perpetuating selective gene knockdown. One technical challenge of siRNA-based gene silencing is that naked siRNA is too large and negatively charged to readily cross the cell membrane and is subjected to rapid degradation by endogenous enzymes (Whitehead et al., 2009). Furthermore, the delivery may not be specific to the target site when using a systemic siRNA delivery method, possibly increasing the risk of off-target effects. To overcome these issues, we used a lipid-based transfection reagent (Oligofectamine^TM^) to improve siRNA delivery and directly administered Bet1L siRNA into the target muscle (Dana et al., 2017; Seven et al., 2018). Liposomes have been recognized as an effective delivery vehicle for a broad spectrum of therapeutics including siRNA (Dana et al., 2017). In this study, we used Oligofectamine that has a proprietary formulation suitable for transferring nucleotides, including siRNA, into cells and tissues in a non-toxic fashion (Nakajima et al., 2012; Seven et al., 2018). Similarly, as we performed in the present study, gene silencing by siRNA has been performed successfully targeting various organ/tissue types such as eyes (Reich et al., 2003; Fattal and Bochot, 2006), mucosal membrane (Palliser et al., 2006), skin (Inoue et al., 2007), and skeletal muscle (Tang et al., 2012).

In this study, intramuscular BET1L siRNA injection sufficiently worked for gene knockdown in rat skeletal muscle. Western blotting and immunohistochemical analyses proved a significantly lower BET1L protein level in the treated TA muscles when compared to control samples. It should be considered that potential adverse reactions or variations might occur by the injection of siRNA and/or the transfection reagent. To secure sufficient comparisons to Bet1L siRNA-treated groups, we prepared three control groups (scrambled siRNA-injected, vehicle only, and no injection).

The knockdown of Bet1L significantly decreased innervation and increased denervation at the NMJs in both WT and ALS rats. These results prove Bet1L has critical contributions in NMJ maintenance. Our study also suggests the idea that the loss of Bet1L at the NMJs likely initiates before NMJ denervation and disease onset occur in ALS rats (Lynch et al., 2021). However, the NMJ denervation following Bet1L gene silencing may not fully implicate significant involvements of this protein as an active contributor to trigger early pathogenesis in ALS because NMJs preserve their plasticity and are constantly remodeling throughout their lifespan (Slater, 2020; Iyer et al., 2021). Interestingly, NMJ innervation was greater after 3 weeks of siRNA injections in both SOD1 and WT rats when compared to the levels in the 1st week of injections. This may also explain a possibility of potential compensatory mechanisms of the NMJ regeneration in response to acute denervation induced by Bet1L gene silencing. To determine this possibility, we would need to evaluate the long-term effects of Bet1L knockdown.

To further characterize possible roles of Bet1L in early ALS disease pathogenesis, we extended the period of Bet1L knockdown in target muscles from 1 to 3 weeks. Based on our results in motor neuron survival, the longer trial of gene silencing sufficiently worked and caused motor neuron degeneration in the lumbar spinal cord segments that have projections to the siRNA-injected muscles. Together, these data serve as additional evidence supporting the idea of the “dying back” phenomenon in ALS pathology: the disease may originate in the peripheral tissues, such as NMJs and skeletal muscle, and a retrograde signaling cascade leads to motor neuron death (Fischer et al., 2004; Krakora et al., 2012; Moloney et al., 2014; Yaron and Schuldiner, 2016; Piol et al., 2023; Shefner et al., 2023; Gao et al., 2024; Kubat and Picone, 2024). Neuromuscular transmission defects and synaptic aberrance have been shown to precede motor neuron degeneration and motor symptoms in rodent (Rocha et al., 2013; Chand et al., 2018) and Drosophila (Shahidullah et al., 2013) models of ALS. Furthermore, skeletal muscle-specific expression of mutant (G93A or G37R) and wild-type human SOD1 in transgenic mice disrupted NMJs and led to motor neuron degeneration and a corresponding functional phenotype (Wong and Martin, 2010; Martin and Wong, 2020).

As a considerable note, the degree of NMJ denervation, motor neuron degeneration, and BBB score reduction was more significant in ALS rats when compared to WT rats. This brings forth the idea that Bet1L knockdown and the ALS mutation synergistically accelerated NMJ denervation and motor neuron degeneration. Although these ALS rats were confirmed as clinically pre-symptomatic at the point of siRNA injection, the environment in skeletal muscle and at the NMJs may have already been influenced by the ALS mutation, resulting in being more susceptible to Bet1L siRNA (or any treatments) in skeletal muscle. For instance, if the process of Bet1L protein downregulation has already been initiated in the pre-symptomatic ALS rats, additional gene silencing could sufficiently make Bet1L protein expression below the biologically required level to maintain NMJ innervation in ALS rats. Alternatively, the ALS mutation may influence the ability of NMJ regeneration or the recovery against the treatments, leading to more severe denervation following Bet1L siRNA injection in ALS rats. While our present results support possible contributions of Bet1L proteins during the process of ALS pathogenesis, further studies would be required to elucidate this issue.

Additionally, our present results highlight the significance of our previous studies using *ex vivo* gene therapy (stem cell-based growth/trophic factor delivery) targeting skeletal muscle in ALS (Suzuki et al., 2008; Krakora et al., 2013; Van Dyke et al., 2016; Suzuki and Svendsen, 2016). We previously demonstrated the therapeutic benefits of neuroprotective trophic factor delivery using genetically modified human stem cells targeting the skeletal muscle to prevent pathology in motor neurons and their NMJs during ALS. Specifically, we delivered a combination of glial cell line-derived neurotrophic factor (GDNF) and/or vascular endothelial growth factor (VEGF) to muscle using human mesenchymal stem cells (hMSCs); the hMSCs survive, synthesize, and release growth factors, and slow disease progression in SOD1^G93A^ rats (Suzuki et al., 2008; Krakora et al., 2013). Together with our current study, it is important to elucidate the contributions of NMJ degeneration in ALS pathogenesis for novel treatment of ALS (Krakora et al., 2012; Cantor et al., 2018; Shefner et al., 2023).

Motor function analysis using the BBB rating scale indicated that Bet1L gene silencing for 3 weeks significantly altered the motor function in the injected limb of ALS rats. The original classifications of the BBB scoring indicate that pre-symptomatic was defined as ranging from 21 to 18, symptom onset from 17 to 14, and symptomatic from 13 to 0 in ALS rodents (Basso et al., 1995). In our study, the change in locomotion consistently showed below 17 in the BBB scoring following Bet1L siRNA injection for 3 weeks. Thus, the disease stage of Bet1L siRNA-injected rats was shifted from pre-symptomatic to symptomatic, initiating disease onset earlier. Obvious NMJ denervation was already identified by Bet1L siRNA injection after 1 week, but no significant changes had yet been observed in the BBB score. These results correspond to the previous findings showing that muscle function changes could occur after NMJ denervation in ALS mice (Clark et al., 2016).

One remaining question is how Bet1L contributes to maintaining NMJ integrity. Although the biological roles of Bet1L remain unknown, currently available literature suggests that Bet1L may aid in the integrity of the Golgi apparatus and regulation of retrograde synthesis through the SNARE protein complex (Xu et al., 2002; Tai et al., 2004). Bet1L was not expressed in motor neuron axons, terminal Schwann cells, or kranocytes (Lynch et al., 2021). Bet1L was focally localized in the laminar area of the NMJs, between the muscle endplate and motor nerve terminal. As vesicle-mediated signaling is necessary to maintain the synapse between motor axon and muscle (Badawi and Nishimune, 2018; Madison and Robinson, 2019; Maggio et al., 2019), it is possible that Bet1L may be involved in retrograde signaling from skeletal muscle for NMJ maintenance and supporting neuronal activity and survival. The unique localization of Bet1L at the NMJs, as opposed to the Golgi apparatus gives rise to a strong potential for Bet1L to have a novel function in maintaining NMJ integrity. Alternatively, Bet1L may be involved in acetylcholine receptor trafficking and clustering as a transporter from skeletal muscle to the NMJs. In ALS, morphological changes of muscle endplates were observed prior to symptom onset (Clark et al., 2016; Slater, 2020).

Another possibility is that Bet1L may serve as a component of extracellular vesicles (exosomes) being released from skeletal muscle. Interestingly, a proteomic database for extracellular vesicle studies (Vesiclepedia, http://www.microvesicles.org) indicates the appearance of Bet1L protein in some preparations of extracellular vesicles (Kalra et al., 2012; Pathan et al., 2019). Extracellular vesicles coming from both neurons and muscle cells have recently been found to have a role in NMJ regulation (Madison and Robinson, 2019; Maggio et al., 2019). In fact, it was recently reported that extracellular vesicles containing dysregulated miRNAs involved in transcription and protein ubiquitination were found in ALS patients (Saucier et al., 2019). Altogether, there seems to be a possibility that Bet1L may work with extracellular vesicles from skeletal muscle, leading to NMJ stability and motor neuron survival (Carata et al., 2024; Sbarigia et al., 2024).

The biological insight into Bet1L is still limited at this moment, but a recent genome-wide associated study (GWAS) proposed a possible link of Bet1L with human lifespan (Akiyama et al., 2023). In this study, the authors reported that a new locus in *BET1L* was associated with survival time in a large cohort of Japanese participants. Furthermore, there was a significant overlap with expression quantitative trait loci (eQTL, genomic loci that explain variation in expression levels of mRNAs) of *BET1L* in skeletal muscle. As well as its implications in ALS, skeletal muscle undergoes a variety of structural and functional changes in normal aging. This may also link to its roles in diseases such as sarcopenia, an age-associated decline in skeletal muscle mass, force, and function (Cruz-Jentoft and Sayer, 2019; Azzolino et al., 2024). When counting the fact that ALS is an age-associated neurodegenerative disorder (Valdez et al., 2012; Pandya and Patani, 2020; Azzolino et al., 2024), interplaying roles of Bet1L in aging and ALS would be valuable topics for our future research.

Although our study demonstrated the importance of Bet1L to NMJ maintenance, it was not easy to explain specific mechanisms behind how the reduced levels of Bet1L played this role. Also, as we mainly used pre-symptomatic ALS rats in the present work, we are not able to answer whether the silencing of the Bet1L gene is sufficient to cause motor neuron death in WT rats. In addition to these WT rat studies, it would also be worthwhile if we could see how Bet1L serves in maintaining NMJ integrity under peripheral nerve damage and neuromuscular disorders, such as spinal muscular atrophy and *myasthenia* gravis, which have been known to link to NMJ degeneration and distal axonopathy in pathology.

## 5 Conclusion

In this study, we demonstrated that Bet1L knockdown induced NMJ denervation, motor dysfunction, and motor neuron death in presymptomatic ALS rats. Our results highlight the importance of Bet1L in NMJs and skeletal muscle in ALS pathogenesis. Future research directions should be explored to define molecular and cellular mechanisms behind how Bet1L contributes to NMJ maintenance. Further, a new evaluation of Bet1L as a promising biomarker in ALS proteins may also be an attractive direction using skeletal muscle and other biological samples from ALS models and patient samples. These contributions would be significant toward finding effective therapeutic strategies targeting skeletal muscle to preserve/restore motor function as well as new disease markers for ALS diagnosis.

## Data Availability

The raw data supporting the conclusions of this article will be made available by the authors, without undue reservation.
